# Double kissing inflation outside the stent secures the patency of small side branch without rewiring

**DOI:** 10.1186/s12872-021-02028-z

**Published:** 2021-05-07

**Authors:** Hongbo Yang, Yanan Song, Jiatian Cao, Xueyi Weng, Feng Zhang, Yuxiang Dai, Hao Lu, Chenguang Li, Zheyong Huang, Juying Qian, Junbo Ge

**Affiliations:** Department of Cardiology, Zhongshan Hospital, Fudan University, Shanghai Institute of Cardiovascular Diseases, Xietu Road No. 1609, Shanghai, 200032 People’s Republic of China

**Keywords:** Coronary bifurcation lesion, Percutaneous coronary intervention, Side branch protection, Kissing balloon inflation

## Abstract

**Background:**

The jailed balloon technique is widely used for coronary bifurcation lesions, but a residual risk of SB occlusion remains, necessitating SB rewiring and further interventions, including balloon inflation or stenting, which may result in failure and SB loss. This study introduced a novel modified technique of small side branch (SB) protection, namely, double kissing inflation outside the stent (DKo) technique, for coronary bifurcations without the need for SB rewiring.

**Methods:**

We performed the DKo technique in consecutive patients in our center from 1/2019 to 12/2019. The procedure was as follows. We inserted a guide wire into both branches followed by proper preparation. The SB balloon was simultaneously inflated with main vessel (MV) stenting. The SB balloon remained in situ until it was kissing inflated with postdilation of the bifurcation core, which is different from traditional strategies. The proximal optimization technique was performed with a short noncompliant balloon strictly not exceeding the bifurcation. Rates of SB loss and in-hospital outcomes were evaluated.

**Results:**

The technique was successfully performed in all 117 enrolled patients without any rewiring or SB loss. The mean lesion lengths of the MV and SB were 38.3 ± 19.9 mm and 11.7 ± 7.1 mm, respectively. On average, 1.5 ± 0.6 stents were used per patient, while the mean pressure of the SB balloon was 7.4 ± 3.1 atm. DKo achieved excellent procedural success in the proximal and distal MVs: increased minimal lumen diameter (0.64 ± 0.58 mm to 3.05 ± 0.38 mm, *p* < 0.001; 0.57 ± 0.63 mm to 2.67 ± 0.35 mm, *p* < 0.001) and low residual stenosis (11.4 ± 3.4%; 7.2 ± 4.6%). DKo secured the patency of the SB without any rewiring and improved the SB stenosis with minimal lumen diameter (0.59 ± 0.48 mm to 1.20 ± 0.42 mm, *p* < 0.001) and stenosis (71.9 ± 19.4% to 42.2 ± 14.0%, *p* < 0.001). No MACE was noted in the hospital.

**Conclusions:**

DKo for bifurcation lesions was shown to be acceptable with high procedural success and excellent SB protection.

## Introduction

Coronary bifurcation lesions are frequently encountered in daily percutaneous coronary intervention (PCI), with a lower rate of procedural success and a higher rate of adverse outcomes [1, 2]. Numerous approaches have been developed to optimize and potentially improve their clinical outcomes [1]. Recent meta-analysis and network meta-analysis showed that DK-crush was associated with fewer major adverse cardiovascular events and device-oriented clinical events in part of bifurcation lesions [3, 4]. However, some randomized comparisons and observational series have shown the superiority of the provisional T-stenting technique with a lower contrast medium volume and X-ray exposure, and shorter fluoroscopy and procedural times [2, 5–7], which is preferred in many bifurcation lesions, and is particularly suitable for small banches [8, 9].

The major problem of the provisional strategy is side branch (SB) occlusion and the resultant worse outcomes, which are caused by plaque shifts, carina shifts, spasms, dissection, protrusion of the stent struts into the SB, and/or changes in the bifurcation angle [10–15]. Wiring SB before main vessel (MV) stenting is associated with the recovery of occluded SB; however, a recovery flow is achieved in only approximately 75% of patients [11, 14, 15]. Therefore, the jailed balloon technique (JBT), a technical innovation of the provisional strategy, was designed to improve the SB patency and greatly reduce SB occlusion due to its higher occupation of balloons at the SB ostium [16–18]. However, SB occlusion sometimes occurs when the MV stent is postdilated after removal of the SB balloon. SB compromise occurs at a rate as high as 9–15% in the JBT after MV stenting [16–18]. Once SB impaired, further rewiring, inflation, and stenting had to be attempt to restore blood flow. Sometimes, it failed and resulted in SB dissection and loss, which necessitated improvement in the traditional JBTs.

In this paper, we propose a novel modified technique of SB protection to prevent the residual risk of SB occlusion after withdrawal of the SB balloons in the traditional JBT. We prolonged the protection of the balloon to postdilation of the bifurcation, as double kissing inflation outside the stent (DKo), to secure the patency of the branch and then carried out a high-quality proximal optimal technique (POT) to ensure good apposition of the main vessel.

## Materials and methods

### Study population

In Fudan University Affiliated Zhongshan Hospital between 1/2019 and 12/2019, patients who presented with non-ST-elevation myocardial infarction or angina pectoris and underwent PCI of de novo coronary bifurcation lesions with a visually estimated diameter stenosis ≥ 70% involving the MV and ≥ 50% involving the ostial SB were analyzed. The vessel size must be ≥ 2.5 mm in the MV and ≤ 2.5 mm in the SB by visual estimation on coronary angiography. SB was at a high risk of occlusion classified as Medina type 1,1,1 or 1,0,1. Baseline characteristics were obtained from the medical records, and bifurcation lesions were classified according to the Medina classification. Patients who underwent the 2-stent technique or other protective strategies were excluded. One hundred and seventeen patients underwent the DKo technique. All patients were consented for the procedure and administrative approval for the retrospective analysis was provided by the local ethics committee.

### Procedure technique

All patients received aspirin (300 mg) and a loading dose of clopidogrel (300 mg) or ticagrelor (180 mg) prior to or at the time of the PCI. During the procedure, unfractionated heparin (UFH) or bivalirudin was administered for anticoagulation. The decision to use DKo was at the operator’s discretion, generally in those with a high risk of SB occlusion, including significant SB ostial disease, difficult bifurcation angles, and/or anatomy and lesion morphology that would make SB access difficult. 6/7Fr guiding catheters were used for the procedure via transradial approaches. The use of intravascular ultrasound (IVUS), as well as any further intervention based on the IVUS findings, was based solely on the operator’s judgment. Following PCI, all patients were monitored for periprocedural complications. Cardiac troponins were measured before the procedure and 12–18 h postprocedure. Marker elevation ≥ 5 times the upper limit of normal was considered significant in patients with normal cardiac markers at baseline. For patients who already had elevated cardiac enzyme levels before the procedure, marker elevation ≥ 20% of the previous value was considered significant [19]. Patients were discharged with standard dual antiplatelet therapy according to the current guidelines.

**Fig. 1:**
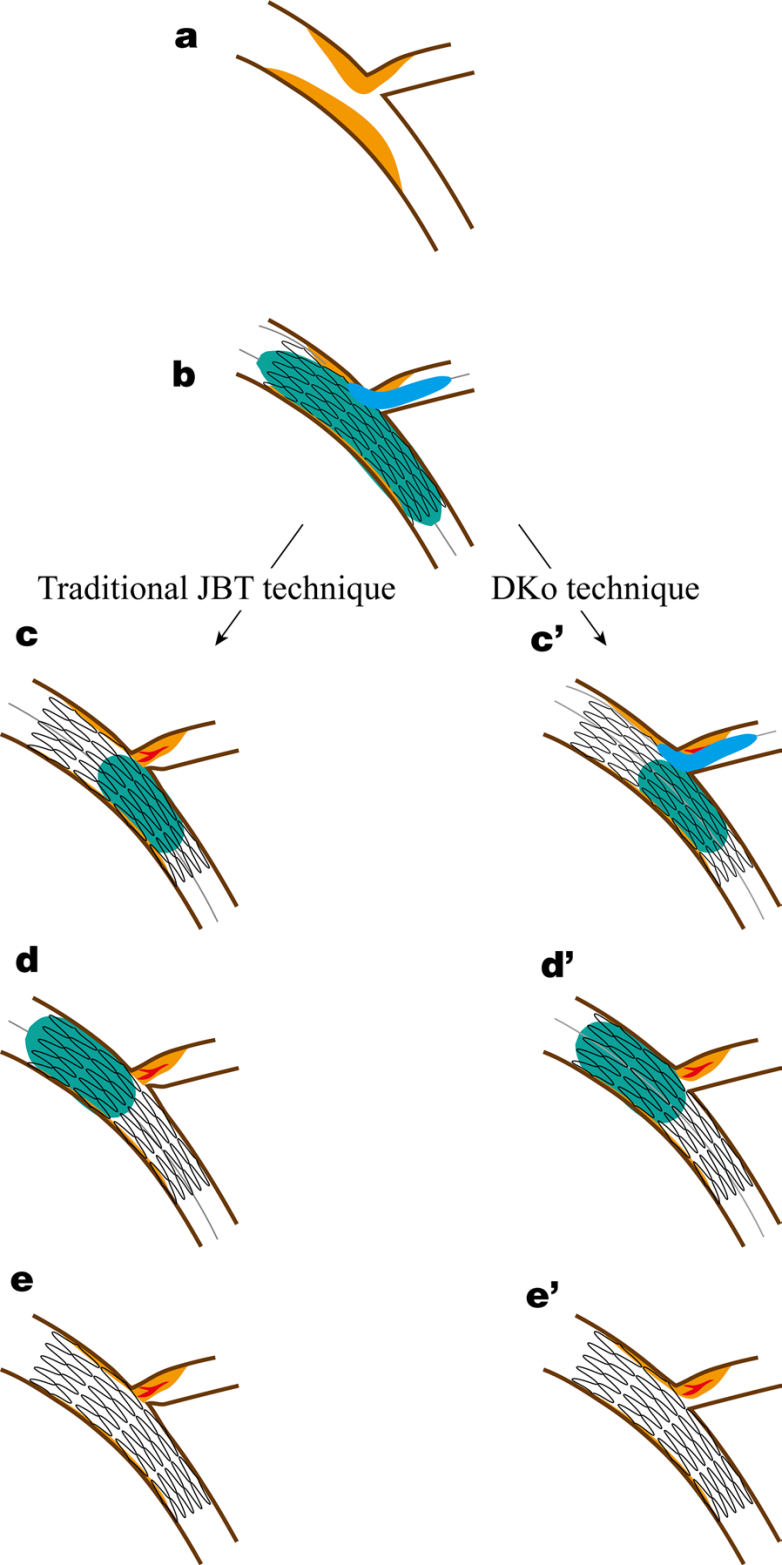
Schematic image depicting the key steps of traditional JBT and DKo techniques. **a** Coronry bifurcation lesion. **b** Stent deployment with jailed balloon protection. **c** Postdilation of bifurcation in traditional JBT caused carina shift. **d** POT, **e** compromise of side branch, **c′** Kissing inflation of bifurcation core. **d′** POT. **e′** Patency of side branch. JBT, jailed balloon technique; DKo, double kissing inflation outside the stent; POT, proximal optimization technique

**Fig. 2:**
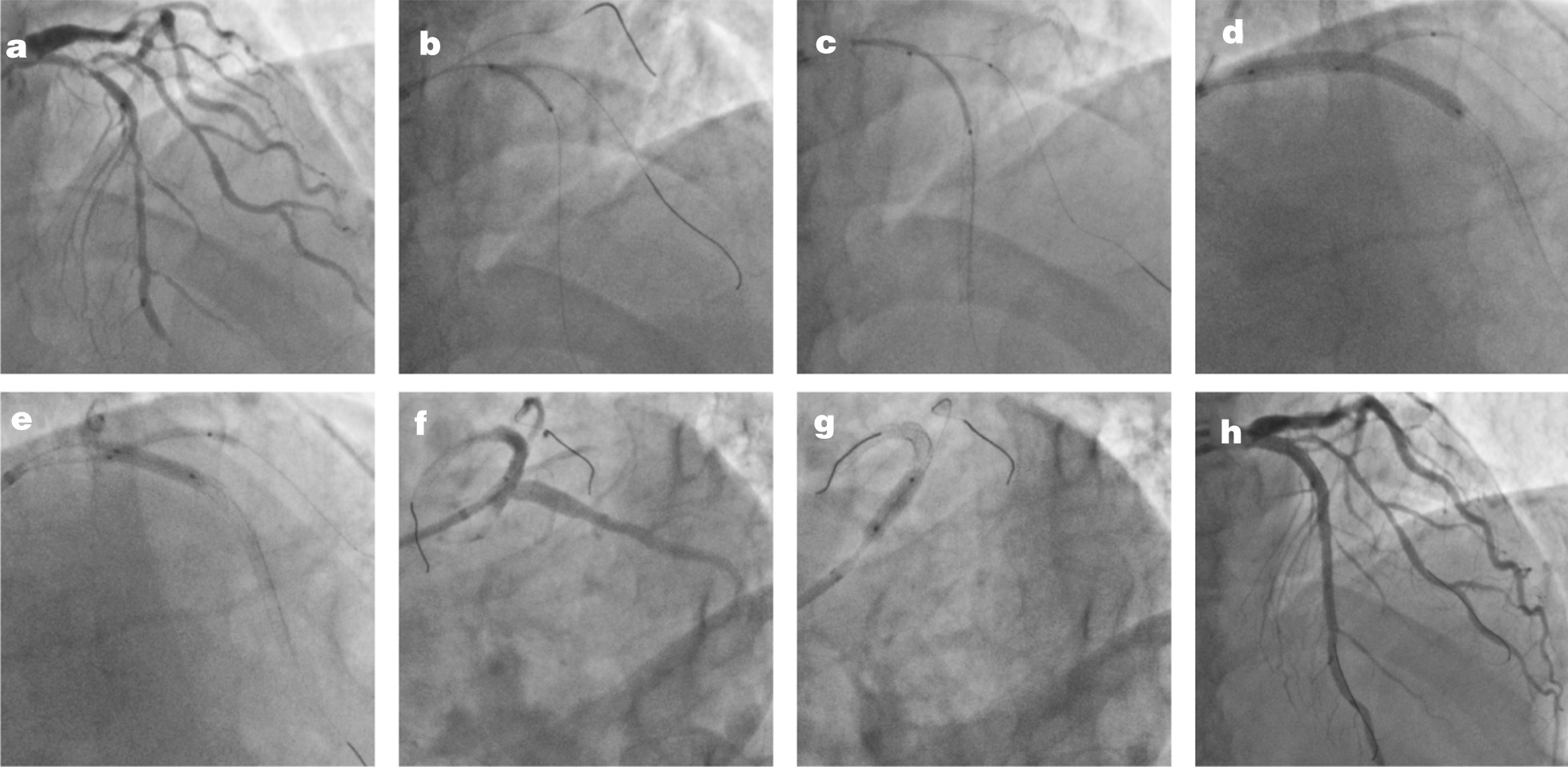
Step-by-step procedures of double kissing inflation outside the stent technique in one representative patient. **a** Bifurcation lesion of left anterior descending artery (LAD) and first diagonal branch (D1). **b** Wiring both branches and management of LAD with a semicompliant balloon; **c** A 3.5 * 38 mm stent was used to cover the LAD lesion, and a 2.0 * 20 mm balloon was delivered into the D1 covering its ostium; **d** First kissing inflation of the stent balloon (12 atm) and balloon (6 atm); **e** Second kissing inflation of D1 balloon (6 atm) and a noncompliant 3.5 * 15 mm balloon at the bifurcation core (20 atm); **f** TIMI 3 flow in D1 and LAD; **g** Proximal optimization technique was performed with a noncompliant 4.0 * 10 mm balloon; **h** Final results

The standardized operation of DKo was developed independently at our institution. The critical mechanisms for SB protection are schematically shown in Fig. 1c′–e′, and the step-by-step procedures are detailed angiographically in Fig. 2. (1) The procedure starts with wiring both branches, and then MV and/or SB lesions are managed with semicompliant balloon inflation as necessary. (2) A stent with optimal size and length is used to cover the MV lesion, and then a proper balloon that is angiographically sized to approximate the SB vessel diameter (generally 1.5–2.5 mm and of adequate length) is introduced into the SB covering the ostium with part of the balloon behind the stent. The SB balloon should not protrude into the MV too much, which should be located at the expansion part of the bifurcation. (3) For the first kissing inflation, the stent balloon and SB balloon are inflated simultaneously for 10–15 s to deploy the stent. The stent is deployed to nominal pressure, while the SB balloon is inflated at the discretion of the operator, usually 6–10 atm according to the diameters of the balloon and vessel. (4) For the second kissing inflation, the SB balloon remaining in situ is inflated simultaneously with the balloon (stent balloon or noncompliant balloon) at the bifurcation core for 10–15 s to optimize stent apposition. (5) The balloon outside the stent is then deflated and removed. (6) POT is performed strictly not exceeding the bifurcation with a short noncompliant balloon to correct stent apposition, which is positioned by stent boost and angiography. It is critical to prevent carina shift and SB compromise in traditional JBT. (7) Angiography is performed to confirm the final results of the bifurcation lesion.

### Definitions and clinical outcomes

Procedural and in-hospital clinical outcomes were recorded. Angiographic outcomes for each index PCI were independently reviewed by an author, who was not an operator in the case, and thrombolysis in myocardial infarction (TIMI) flow grading was established for both MV and SB. SB loss was defined as TIMI 0–1 flow immediately following MV stenting. Quantitative coronary analysis (QCA) was performed at baseline and post PCI by standard techniques with automated edge-detection algorithms (CASS-5.2, Pie Medical, Maastricht, The Netherlands). Bifurcation lesions were analyzed in 3 segments: proximal MV, distal MV and SB. According to the algorithm in the dedicated software, reference vessel diameter, minimal lumen diameter and diameter stenosis were measured. Procedure success was defined as successful implantation of the stent and final residual stenosis ≤ 30% without flow impairment or complication. The definition of procedure success includes two parts. The first part is successful stent implantation with final residual stenosis < 30%. The other one is unimpaired flow, which means TIMI 3 flow in the end or TIMI 2 flow in patients presented as coronary slow flow in other coronary arteries being not intervened. The procedural time was defined from “guidewire in” to “guidewire out.” IVUS imaging was performed after intracoronary administration of 0.1–0.2 mg nitroglycerin, and quantitative measurements included the external elastic membrane (EEM) and minimal stent area (MSA). The stent symmetry index (SSI) was calculated by dividing the minimal stent diameter by the maximal stent diameter at a cross-section with the smallest lumen cross-sectional area in the proximal, bifurcation, and distal stent segments, as previously described. Postprocedural incomplete stent apposition (ISA) was defined as lack of contact between at least one strut and the underlying arterial wall intima that did not overlap a side branch with evidence of blood flow behind the strut [20–22]. Major adverse cardiac events (MACEs), as a composite of cardiac death, myocardial infarction (MI), and target lesion revascularization (TLR), were noted in the hospital.

### Statistical analysis

Data are presented as the mean ± standard deviation or frequency (percentage). Continuous variables were analyzed using Student's t test, while categorical data were analyzed using the χ^2^ test. A *p* value < 0.05 was considered to be statistically significant. SPSS (IBM, SPSS Statistics, 22 version) was used.

## Results

### Baseline demographics

DKo was successfully performed in all 117 enrolled patients with true bifurcation lesions according to the standardized protocol. The baseline characteristics are summarized in Table 1. Of them, 105 (89.7%) patients were male, with a mean age of 64 ± 10 years old. The clinical profile showed a high percentage of patients with a prior PCI history (39.3%), with the expected prevalence of hypertension (63.0%), diabetes (29.1%), hyperlipidemia (13.7%), and current smokers (27.4%). The indication for PCI was non-ST-elevation myocardial infarction in 19 cases and stable angina in 98 cases. All patients successfully underwent stent implantation and were discharged with dual antiplatelet drugs and statin therapy. Of them, 90 (76.9%) patients received beta-blockers, while 76 (65.0%) patients received renin-angiotensin system inhibitors.

**Table 1:** Clinical characteristics (n = 117)

Age, y	64.0 ± 10.3
Male (%)	105 (89.7)
*Medical history*	
Hypertension (%)	74 (63.2)
Diabetes (%)	34 (29.1)
Hyperlipidemia (%)	16 (13.7)
Smoking (%)	32 (27.4)
Prior PCI (%)	46 (39.3)
Prior CABG (%)	9 (7.7)
Myocardial infarction history (%)	24 (20.5)
*Clinical manifestation*	
Stable angina (%)	98 (83.8)
Acute coronary syndrome (%)	19 (16.2)
LVEF (%)	60.8 ± 6.6
*Antithrombotic therapy during intervention*	
Heparin (%)	113 (96.6)
Bivalirudin (%)	4 (3.4)
Glycoprotein IIb/IIIa inhibitor (%)	14 (12.0)
*Discharge medications*	
Aspirin (%)	105 (89.7)
Cilostazol (%)	12 (10.3)
Clopidogrel (%)	82 (70.1)
Ticagrelor (%)	35 (29.9)
Beta blocker (%)	90 (76.9)
RAS inhibitors (%)	76 (65.0)
Statin (%)	117 (100.0)
Ezetimibe (%)	12 (10.3)

Data are presented as mean ± SD or n (%)

PCI, percutaneous coronary intervention; LVEF, left ventricular ejection fraction; RAS inhibitors, renin-angiotensin-system inhibitors

### Procedural characteristics

**Fig. 3:**
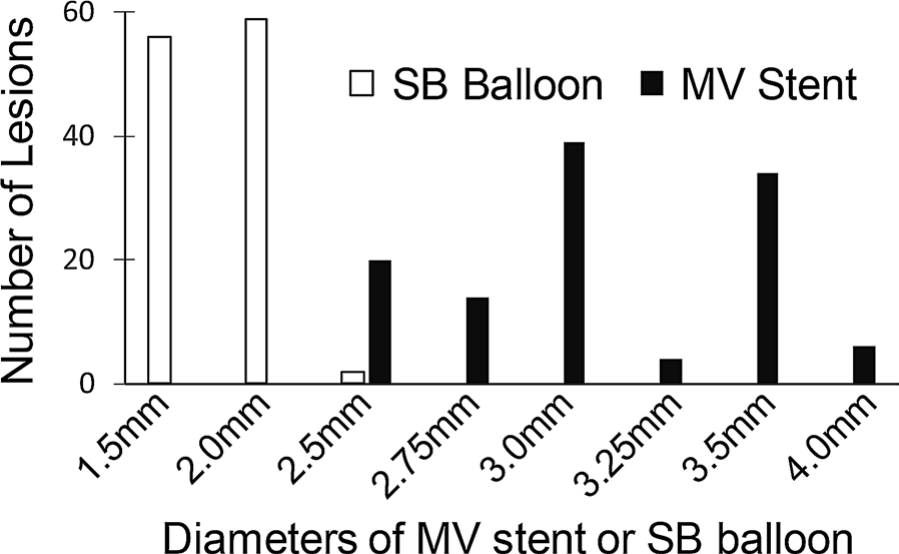
Distribution of main vessel stent and side branch balloon diameters. This is distribution of main vessel stent and side branch balloon diameters

The angiographic and procedural characteristics are shown in Table 2. The majority (89.7%) of patients had more than one diseased vessel. Treated bifurcations were located at a variety of bifurcations, and most (98.3%) of them were Medina 1.1.1 type bifurcation lesions. The bifurcation angle was Y type (< 70°) in 71 patients, and calcification occurred in the MV of 26 patients. Procedures were all performed via a transradial approach with 6F (98.3%) or 7F (1.7%) guiding catheters. The mean lesion lengths of the MV and SB were 38.3 ± 19.9 mm and 11.7 ± 7.1 mm, respectively. Before the intervention, TIMI 3 flow in the MV and SB was observed in 91 (77.8%)  and 97 (82.9%) patients, respectively. On average, 1.5 ± 0.6 stents were used per patient, and the mean pressure of the SB balloon was 7.4 ± 3.1 atm. The diameters of the MV stent and SB balloon in DKo were 3.1 ± 0.4 mm and 1.8 ± 0.3 mm, respectively. The distribution of the MV stent and SB balloon diameters is shown in Fig. 3. The diameter of the most frequently used balloons was 2.0 mm, while the majority of stents were 3.0 mm and 3.5 mm.

**Table 2:** Angiographic and procedural characteristics (n = 117)

*Number of diseased vessel*	
One vessel (%)	12 (10.3)
Two vessels (%)	24 (20.5)
Three vessels (%)	81 (69.2)
*Target bifurcation*	
Left main coronary artery (%)	18 (15.4)
LAD-branch (%)	69 (59.0)
LCX-branch (%)	22 (18.8)
RCA-branch (%)	8 (6.8)
*Medina classification*	
Medina 1.1.1 (%)	115 (98.3)
Medina 1.0.1 (%)	2 (1.7)
*Bifurcation angle* (°)	
< 70 (%)	71 (60.7)
70–90 (%)	28 (23.9)
> 90 (%)	4 (3.4)
*Calcification*	
No (%)	91 (77.8)
Mild-moderate (%)	16 (13.7)
Severe (%)	10 (8.5)
*Guiding catheter size*	
6 French (%)	115 (98.3)
7 French (%)	2 (1.7)
MV lesion length (mm)	38.3 ± 19.9
SB lesion length (mm)	11.7 ± 7.1
*MV pretreatment TIMI flow*	
0–1 (%)	22 (18.8)
2 (%)	4 (2.5)
3 (%)	91 (77.8)
*SB pretreatment TIMI flow*	
0–1 (%)	16 (13.3)
2 (%)	4 (2.5)
3 (%)	97 (82.9)
SB predilation	12 (10.3)
*DKo details*	
Number of stents	1.5 ± 0.6
MV stent diameter (mm)	3.1 ± 0.4
MV stent length (mm)	43.6 ± 22.3
SB balloon diameter (mm)	1.8 ± 0.3
SB balloon length (mm)	16.2 ± 2.3
SB balloon pressure (atm)	7.4 ± 3.1
Procedural time (min)	18.7 ± 5.0

Data are presented as mean ± SD or n (%)

LAD, left anterior descending coronary artery; LCX, left circumflex coronary artery; Dg, diagonal branch; MV, main vessel; SB, side branch; TIMI, thrombolysis in myocardial infarction; DKo, double kissing inflation outside the stent; atm, atmosphere

### Quantitative coronary analysis

The QCA analysis is displayed in Table 3. DKo achieved excellent procedural success in the proximal and distal MVs: increased minimal lumen diameter (0.64 ± 0.58 mm to 3.05 ± 0.38 mm, *p* < 0.001; 0.57 ± 0.63 mm to 2.67 ± 0.35 mm, *p* < 0.001) and low residual stenosis (11.4 ± 3.4%; 7.2 ± 4.6%). Meanwhile, DKo improved SB stenosis with an increased minimal lumen diameter (0.59 ± 0.48 mm to 1.20 ± 0.42 mm, *p* < 0.001) and reduced stenosis (71.9 ± 19.4% to 42.2 ± 14.0%, *p* < 0.001).

**Table 3:** Quantitative coronary angiographic analysis (n = 117)

	Baseline	Postprocedure	*p* value
*Proximal main vessel*			
RVD (mm)	3.36 ± 0.42	3.53 ± 0.41	< 0.001
MLD (mm)	0.64 ± 0.58	3.05 ± 0.38	< 0.001
Diameter stenosis (%)	81.6 ± 15.5	11.4 ± 3.4	< 0.001
*Distal main vessel*			
RVD (mm)	2.87 ± 0.37	2.88 ± 0.38	0.311
MLD (mm)	0.57 ± 0.63	2.67 ± 0.35	< 0.001
Diameter stenosis (%)	82.7 ± 17.6	7.2 ± 4.6	< 0.001
*Side branch*			
RVD (mm)	2.00 ± 0.38	2.05 ± 0.39	< 0.001
MLD (mm)	0.59 ± 0.48	1.20 ± 0.42	< 0.001
Diameter stenosis (%)	71.9 ± 19.4	42.2 ± 12.5	< 0.001

Data are presented as mean ± SD or n (%)

RVD, reference vessel diameter; MLD, minimal lumen diameter

### IVUS analysis

**Fig. 4:**
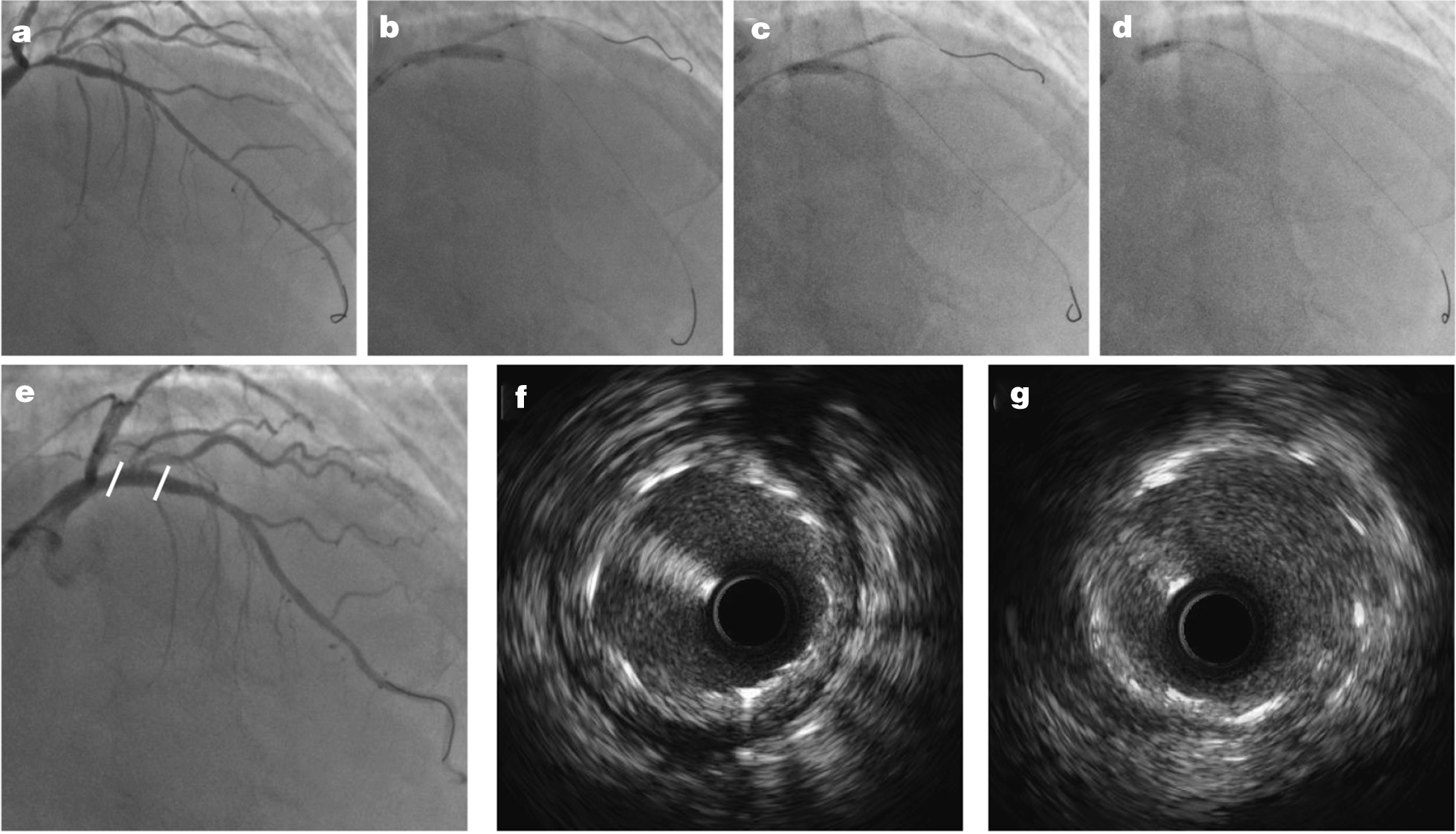
Representative images of one patient. **a** Bifurcation lesion of left anterior descending artery (LAD) and first diagonal branch (D1). **b** First kissing inflation of the stent balloon (12 atm) and balloon (6 atm); **c** Second kissing inflation of D1 balloon (6 atm) and a noncompliant 3.5 * 15 mm balloon at the bifurcation core (20 atm); **d** Proximal optimization technique; **e** Final coronary angiogram and its complete stent apposition and good stent symmetry in both distal (**g**) and proximal (**h**) stent segments

The results of the IVUS analysis in 21 patients after the DKo procedure in the proximal and distal segments are shown in Table 4. The balloon diameter, external elastic membrane, and minimal stent area in the proximal segment were larger than those in the distal segment. In the proximal and distal segments, the stent was symmetrically expanded, and the percentages of SSI were not significantly different. Incomplete stent apposition was observed in the proximal stent segment of 3 patients and in the distal stent segment of 2 patients, which also showed no significant differences. Representative images are displayed in Fig. 4.

**Table 4:** Intravascular ultrasound analysis (n = 21)

Stent diameter (mm)	3.25 ± 0.38
Stent length (mm)	30.3 ± 4.7
*Proximal main vessel*	
Proximal optimal technique balloon size (mm)	3.61 ± 0.38
External elastic membrane (mm^2^)	18.3 ± 4.1
Minimal stent area (mm^2^)	7.94 ± 1.78
Stent symmetry index	0.89 ± 0.03
Incomplete stent apposition (%)	3 (14.3)
*Distal main vessel*	
Post-stent dilation balloon size (mm)	3.19 ± 0.33
External elastic membrane (mm^2^)	12.7 ± 3.7
Minimal stent area (mm^2^)	6.01 ± 1.75
Stent symmetry index	0.88 ± 0.08
Incomplete stent apposition (%)	2 (9.5)

Data are presented as mean ± SD

### Acute procedural and clinical outcomes

Immediate procedural and clinical outcomes are presented in Table 5. DKo was successfully performed in all enrolled patients without any rewiring SB. Periprocedural cardiac biomarkers increased in one patient without discomfort or electrocardiographic changes. No SB was lost, but 1 patient showed TIMI 2 flow in the first diagonal branch, who showed TIMI 2 flow in the left anterior descending,left circumflex and right coronary artery. No stent edge dissection occurred, but dissection of the SB ostium developed in 1 patient. Further intervention was not performed due to its TIMI 3 flow. No MACE occurred in the hospital. The jailed balloon and wire were successfully removed without any damage or entrapment.

**Table 5:** Clinical outcomes

Procedure success (%)	117 (100.0)
Periprocedural cardiac biomarker increase (%)	1 (0.9)
In-hospital MACE (%)	0 (0.0)
Post TIMI 3 flow (%)	116 (99.1)
Temporal SB occlusion (%)	0 (0.0)
Side branch loss (%)	0 (0.0)
*SB TIMI flow after MV stenting*	
0–1 (%)	0 (0.0)
2 (%)	1 (0.9)
3 (%)	116 (99.1)
*Dissection*	
Proximal stent edge (%)	0 (0.0)
Distal stent edge (%)	0 (0.0)
SB ostium (%)	1 (0.9)
SB stenting	0 (0.0)
Balloon or wire entrapment	0 (0.0)

Data are presented as n (%)

MACE, major adverse cardiovascular events; MV, main vessel; SB, side branch; TIMI, thrombolysis in myocardial infarction

## Discussion

This study showed that DKo, a novel modified technique of SB protection without SB rewiring, was successfully applied in coronary bifurcation with high procedural success. DKo secured excellent patency of the SB without the need for rewiring. DKo was well performed in bifurcation lesions without MACE, balloon damage or entrapment.

JBT was first reported by Burzotta et al., and it represents a significant evolution in the protection of SB [16]. Traditional JBT results in temporary SB compromise in 9–15% of patients with a small profile of jailed balloons [16–18], and a variety of modified JBTs have been adopted to improve the patency of SB [23–27]. However, SB loss sometimes occurs when postdilating during removal of the SB balloon. There are two major reasons, one of which is plaque shift and/or carina shift caused by postdilation of the stent similar to wire protection [18, 24], and the other is dissection and occlusion of the SB during rewiring. The DKo approach, reported in this study, secures the patency of the SB without the need for rewiring to perform a provisional strategy and it has some advantages: (1) We prolonged the protection of the inflated balloon to postdilate the bifurcation to secure 100% patency of the branch. Carina shift and plaque shift are the major mechanisms of SB occlusion [11]. DKo pushed the vessel walls outward, during which the first kissing inflation maintained the patency of the SB during MV stenting, and the second kissing inflation prevented SB loss in postdilation, unlike in JBT [18, 24]. This provides better technical support for the implementation and promotion of a single-stent strategy. (2) Long-term inflation of balloons in SB is beneficial to optimize the effect of PCI and reduce the incidence of dissection [28–30]. (3) There is no need for rewiring, which is technically challenging, and can reduce the difficulty of the procedure, shorten the procedure time, and reduce the exposure to radiation and contrast media [23]. (4) DKo does not impact the stent apposition and stent symmetry index in either the proximal or distal stent segments. Therefore, this technology is worth popularizing and applying.

There are some technical issues that need to be noted: (1) The first concern is the location and size of the SB balloon. As kissing inflated balloons are located outside the stent, the risk of proximal dissection theoretically increases. The SB balloon should not protrude into the MV too much, which should preferably be located at the expansion part of the bifurcation. The SB balloon is selected according to the diameter of the SB, whose diameter is generally 1.5–2.5 mm, and the pressure is approximately 6–10 atm. (2) The inflation duration of the SB balloon is another concern. Although long-term balloon inflation is beneficial to optimize the dilatation effect of SB, some patients cannot tolerate continuous flow blockage. The SB balloon can be deflated between the two kissing inflations. (3) Although no balloon entrapment occurred in our study, we suggest that the balloon in the stent should not cross the bifurcation too much during the second kissing inflation to decrease the withdrawal resistance and friction between the SB balloon and stent. (4) POT strictly not exceeding the bifurcation is critical to avoid potential SB compromise without balloon occupation. Stent boost and angiography are used for accurate positioning.

The major concerns about DKo are entrapment of the SB balloon under the MV stent and the possible risk of MV stent strut distortion/malapposition in the MV proximal segment. The proximal MV diameter is larger than the distal MV, and the stent size should be selected according to the distal MV diameter in the provisional strategy [15]. After the SB balloon is inflated outside the stent with stent deployment and postdilation, underexpansion of the stent, which potentially occurs in the proximal MV segment, facilitates the removal of the SB balloon [12, 18]. In addition, calcific bifurcations can potentially cause device entrapment especially if the SB balloon is next to calcium and stent. No balloon entrapment occurred in our study, similar to prior studies concerning JBT [16–18, 25–27]. Nevertheless, we recommend not using force to avoid balloon damage when encountering difficulty in removing the balloons. POT is recommended to ensure appropriate apposition of the stent struts to the vessel wall after removing the SB balloon, which has been illustrated in bench tests and clinical use by intravascular imaging in this and other studies [16, 23]. The study focused on bifurcation lesions of Medina type 1,1,1 or 1,0,1, in which SB had a tendency to occlude. Prevention was more important than rescue to secure the patency of SB.

### Study limitations

There are several limitations of this study. It is a retrospective, observational single-center experience. In addition, calcific bifurcations have higher probability of SB occlusion [31] and potentially cause device entrapment [16–18] especially if the SB balloon is next to calcium and stent. In our experience, there was no SB occlusion or device entrapment in DKo procedure, which might be due to the small sample size. Operators should pay more attention to avoid device entrapment of DKo procedure when it is used in severe calcific lesions. This study does not directly compare DKo with other techniques and only reports the technical feasibility, safety, and early outcomes. The long-term outcomes of DKo require further controlled study with a larger population.

## Conclusions

We introduced a novel modified technique of SB protection for coronary bifurcation lesions without rewiring, which was advantageous to popularize the provisional technique and simplify the treatment of coronary bifurcation lesions.

## Data Availability

The datasets used and/or analyzed during the current study are available from the corresponding author on reasonable request.

## Abbreviations

MV: Main vessel
SB: Side branch
PCI: Percutaneous coronary intervention
DKo: Double kissing inflation outside the stent
JBT: Jailed balloon technique
QCA: Quantitative coronary angiography
IVUS: Intravenous ultrasound
TIMI: Thrombolysis in myocardial infarction
POT: Proximal optimal technique
